# Supplementary material for: Modeling glioblastoma heterogeneity as a dynamic network of cell states
Source: Mol Syst Biol. 2021 Sep 16;17(9):e10105. doi: 10.15252/msb.202010105 (PMC8444284; doi:10.15252/msb.202010105)
Supplement: Supplementary file 6 — Source Data for Figure 5 [file MSB-17-e10105-s004.zip › Figure5A_sourcedata/GSEA_3017/nosigdb_stateE.GseaPreranked.1622795031093/RICHARDS_NOWAKOWSKI_SCIENCE_2017_U1_UPREG.html]

Details for gene set RICHARDS\_NOWAKOWSKI\_SCIENCE\_2017\_U1\_UPREG[GSEA]

|  || Dataset | state63017 |
| Phenotype | NoPhenotypeAvailable |
| Upregulated in class | na\_pos |
| GeneSet | RICHARDS\_NOWAKOWSKI\_SCIENCE\_2017\_U1\_UPREG |
| Enrichment Score (ES) | 0.53218514 |
| Normalized Enrichment Score (NES) | 4.1677403 |
| Nominal p-value | 0.0 |
| FDR q-value | 0.0 |
| FWER p-Value | 0.0 |
Table: GSEA Results Summary

  

Fig 1: Enrichment plot: RICHARDS\_NOWAKOWSKI\_SCIENCE\_2017\_U1\_UPREG      
 Profile of the Running ES Score & Positions of GeneSet Members on the Rank Ordered List

  

| PROBE | GENE SYMBOL | GENE\_TITLE | RANK IN GENE LIST | RANK METRIC SCORE | RUNNING ES | CORE ENRICHMENT || 1 | PMP22 |  |  | 0 | 1.393 | 0.0285 | Yes |
| 2 | CTGF |  |  | 2 | 1.276 | 0.0535 | Yes |
| 3 | CYR61 |  |  | 3 | 1.169 | 0.0774 | Yes |
| 4 | IGFBP5 |  |  | 4 | 1.015 | 0.0982 | Yes |
| 5 | CCL2 |  |  | 5 | 0.975 | 0.1182 | Yes |
| 6 | ID3 |  |  | 6 | 0.972 | 0.1381 | Yes |
| 7 | LRRN3 |  |  | 10 | 0.943 | 0.1538 | Yes |
| 8 | ANXA1 |  |  | 12 | 0.826 | 0.1695 | Yes |
| 9 | CD44 |  |  | 14 | 0.806 | 0.1848 | Yes |
| 10 | CEBPD |  |  | 15 | 0.791 | 0.2010 | Yes |
| 11 | ANXA2 |  |  | 18 | 0.733 | 0.2136 | Yes |
| 12 | PDGFA |  |  | 20 | 0.729 | 0.2274 | Yes |
| 13 | PRSS23 |  |  | 21 | 0.722 | 0.2421 | Yes |
| 14 | LGALS3 |  |  | 25 | 0.697 | 0.2528 | Yes |
| 15 | IFITM3 |  |  | 26 | 0.682 | 0.2668 | Yes |
| 16 | GJA1 |  |  | 33 | 0.618 | 0.2723 | Yes |
| 17 | RAB31 |  |  | 35 | 0.598 | 0.2833 | Yes |
| 18 | EMP1 |  |  | 40 | 0.584 | 0.2905 | Yes |
| 19 | CALD1 |  |  | 41 | 0.580 | 0.3024 | Yes |
| 20 | MEG3 |  |  | 42 | 0.562 | 0.3139 | Yes |
| 21 | PMEPA1 |  |  | 44 | 0.544 | 0.3238 | Yes |
| 22 | IFITM2 |  |  | 46 | 0.536 | 0.3336 | Yes |
| 23 | ARID5B |  |  | 47 | 0.533 | 0.3445 | Yes |
| 24 | S100A10 |  |  | 51 | 0.527 | 0.3517 | Yes |
| 25 | PEG10 |  |  | 54 | 0.506 | 0.3597 | Yes |
| 26 | MRC2 |  |  | 56 | 0.505 | 0.3688 | Yes |
| 27 | MMP2 |  |  | 57 | 0.503 | 0.3791 | Yes |
| 28 | WLS |  |  | 65 | 0.472 | 0.3804 | Yes |
| 29 | HSPA5 |  |  | 73 | 0.449 | 0.3812 | Yes |
| 30 | SERPINB6 |  |  | 77 | 0.440 | 0.3867 | Yes |
| 31 | COL5A2 |  |  | 83 | 0.434 | 0.3896 | Yes |
| 32 | SIPA1L1 |  |  | 84 | 0.429 | 0.3983 | Yes |
| 33 | ATF3 |  |  | 101 | 0.397 | 0.3873 | Yes |
| 34 | CD99 |  |  | 107 | 0.389 | 0.3893 | Yes |
| 35 | TPM1 |  |  | 108 | 0.388 | 0.3972 | Yes |
| 36 | LGALS1 |  |  | 113 | 0.368 | 0.4000 | Yes |
| 37 | MAP4 |  |  | 115 | 0.367 | 0.4063 | Yes |
| 38 | CLU |  |  | 119 | 0.364 | 0.4102 | Yes |
| 39 | PCDH9 |  |  | 121 | 0.359 | 0.4163 | Yes |
| 40 | FNDC3B |  |  | 123 | 0.354 | 0.4224 | Yes |
| 41 | AHNAK |  |  | 125 | 0.351 | 0.4284 | Yes |
| 42 | SEMA3A |  |  | 128 | 0.349 | 0.4331 | Yes |
| 43 | DSTN |  |  | 129 | 0.348 | 0.4403 | Yes |
| 44 | ARL6IP5 |  |  | 134 | 0.345 | 0.4425 | Yes |
| 45 | SOCS3 |  |  | 141 | 0.342 | 0.4424 | Yes |
| 46 | CETN2 |  |  | 145 | 0.338 | 0.4457 | Yes |
| 47 | PDLIM2 |  |  | 155 | 0.329 | 0.4417 | Yes |
| 48 | ZFP36L2 |  |  | 162 | 0.324 | 0.4411 | Yes |
| 49 | SPATS2L |  |  | 163 | 0.323 | 0.4477 | Yes |
| 50 | FZD7 |  |  | 164 | 0.323 | 0.4543 | Yes |
| 51 | DCBLD2 |  |  | 165 | 0.322 | 0.4609 | Yes |
| 52 | JUN |  |  | 172 | 0.316 | 0.4602 | Yes |
| 53 | MBNL2 |  |  | 173 | 0.316 | 0.4667 | Yes |
| 54 | S100A6 |  |  | 176 | 0.312 | 0.4707 | Yes |
| 55 | PLIN3 |  |  | 192 | 0.301 | 0.4589 | Yes |
| 56 | FLNA |  |  | 197 | 0.298 | 0.4602 | Yes |
| 57 | RUNX2 |  |  | 199 | 0.296 | 0.4651 | Yes |
| 58 | CAST |  |  | 202 | 0.294 | 0.4687 | Yes |
| 59 | DKK3 |  |  | 205 | 0.293 | 0.4723 | Yes |
| 60 | SAT1 |  |  | 206 | 0.291 | 0.4782 | Yes |
| 61 | GNG11 |  |  | 210 | 0.289 | 0.4806 | Yes |
| 62 | GNG12 |  |  | 212 | 0.287 | 0.4853 | Yes |
| 63 | MEST |  |  | 215 | 0.285 | 0.4887 | Yes |
| 64 | FERMT2 |  |  | 219 | 0.283 | 0.4909 | Yes |
| 65 | SAMD4A |  |  | 224 | 0.281 | 0.4919 | Yes |
| 66 | SDC2 |  |  | 226 | 0.280 | 0.4964 | Yes |
| 67 | NPAS3 |  |  | 227 | 0.280 | 0.5022 | Yes |
| 68 | PRRX1 |  |  | 229 | 0.278 | 0.5067 | Yes |
| 69 | MEG8 |  |  | 230 | 0.278 | 0.5123 | Yes |
| 70 | GUK1 |  |  | 231 | 0.277 | 0.5180 | Yes |
| 71 | CDK6 |  |  | 238 | 0.269 | 0.5163 | Yes |
| 72 | EHD2 |  |  | 240 | 0.267 | 0.5206 | Yes |
| 73 | CSRP2 |  |  | 247 | 0.264 | 0.5188 | Yes |
| 74 | CKAP4 |  |  | 250 | 0.262 | 0.5218 | Yes |
| 75 | TRAM2 |  |  | 256 | 0.259 | 0.5211 | Yes |
| 76 | GSTM3 |  |  | 257 | 0.259 | 0.5264 | Yes |
| 77 | LEPROT |  |  | 259 | 0.259 | 0.5305 | Yes |
| 78 | WWTR1 |  |  | 263 | 0.256 | 0.5322 | Yes |
| 79 | CHPF |  |  | 269 | 0.251 | 0.5313 | No |
| 80 | ZFP36L1 |  |  | 287 | -0.255 | 0.5162 | No |
| 81 | ZFAS1 |  |  | 308 | -0.260 | 0.4976 | No |
| 82 | AHCY |  |  | 317 | -0.262 | 0.4934 | No |
| 83 | CA12 |  |  | 325 | -0.263 | 0.4904 | No |
| 84 | YME1L1 |  |  | 327 | -0.264 | 0.4946 | No |
| 85 | HSPD1 |  |  | 329 | -0.264 | 0.4988 | No |
| 86 | IMPDH2 |  |  | 351 | -0.269 | 0.4792 | No |
| 87 | STAG1 |  |  | 360 | -0.271 | 0.4751 | No |
| 88 | RECQL |  |  | 390 | -0.279 | 0.4461 | No |
| 89 | GDI2 |  |  | 401 | -0.283 | 0.4400 | No |
| 90 | NASP |  |  | 410 | -0.285 | 0.4362 | No |
| 91 | CDK2 |  |  | 422 | -0.290 | 0.4290 | No |
| 92 | EGFR |  |  | 427 | -0.291 | 0.4302 | No |
| 93 | SEPT10 |  |  | 431 | -0.293 | 0.4326 | No |
| 94 | PNRC2 |  |  | 461 | -0.304 | 0.4041 | No |
| 95 | EPB41L2 |  |  | 488 | -0.310 | 0.3793 | No |
| 96 | PRDX3 |  |  | 489 | -0.310 | 0.3856 | No |
| 97 | GARS |  |  | 506 | -0.316 | 0.3729 | No |
| 98 | DOCK10 |  |  | 520 | -0.320 | 0.3639 | No |
| 99 | SESN3 |  |  | 526 | -0.322 | 0.3645 | No |
| 100 | ING1 |  |  | 541 | -0.329 | 0.3545 | No |
| 101 | SERPINE2 |  |  | 585 | -0.351 | 0.3102 | No |
| 102 | SNHG5 |  |  | 605 | -0.360 | 0.2948 | No |
| 103 | MTHFD2 |  |  | 625 | -0.372 | 0.2797 | No |
| 104 | PID1 |  |  | 645 | -0.385 | 0.2648 | No |
| 105 | ZEB1 |  |  | 653 | -0.396 | 0.2645 | No |
| 106 | TMEM106C |  |  | 654 | -0.397 | 0.2727 | No |
| 107 | EGR1 |  |  | 656 | -0.398 | 0.2796 | No |
| 108 | MAP3K1 |  |  | 702 | -0.431 | 0.2346 | No |
| 109 | MMP16 |  |  | 723 | -0.454 | 0.2199 | No |
| 110 | NUCKS1 |  |  | 744 | -0.471 | 0.2056 | No |
| 111 | GAS1 |  |  | 839 | -0.671 | 0.1068 | No |
| 112 | PDGFRA |  |  | 920 | -0.986 | 0.0311 | No |
Table: GSEA details [plain text format]

  

Fig 2: RICHARDS\_NOWAKOWSKI\_SCIENCE\_2017\_U1\_UPREG: Random ES distribution      
 Gene set null distribution of ES for **RICHARDS\_NOWAKOWSKI\_SCIENCE\_2017\_U1\_UPREG**
